# **Complex Patients; Pharmacists Take the Leading Role in Community**

**Published:** 2013

**Authors:** Farshad Hosseini Shirazi

In daily life, complexity results from countless factors. Culture, environment, social and economic status and physical ability all contribute, and all complicate managing ill health. Pharmacists bear witness to many of these complexities and increasingly must care for what have come to be known as ‘complex patients’. 

The issue of ‘complex patients’ is one that has been addressed by healthcare professionals for some time, yet as the general population in Iran will age in coming years and chronic disease will become more prevalent, it will be an issue that may escalate. It is difficult to define a complex patient, yet it is generally understood that the term applies to those who require an extra amount of care and consideration as a consequence of complicated and extensive medicine regimes compounded by physical and mental limitations. Current global statistics suggest that complex patients comprise upwards of 25% of individuals in primary care practices who fulfil one or more of the following criteria: a) Multiple, well-defined chronic illness with various complications, b) Highly treated, involving invasive procedures, both for diagnosis and therapy, c) A peculiar combination of resiliency and fragility, d) Unexpected responses to common medications and minor illnesses, and e) Longevity (living highly functional lives into the ages of 80’s and 90’s)

These patients are a group of individuals that benefit from the extensive knowledge on medicines that pharmacists possess. As the most accessible healthcare provider, pharmacists come into continual contact with complex patients in the community, putting them at the front line of care with regard to managing many issues that may or may not be medicines-related. With complex patient management demanding an ever-increasing multi-disciplinary approach, the pharmacist›s main focus is medicines management in the face of other complexities. 

With the establishment of the clinical pharmacy residency program, a practice shift that truly put pharmacists at the front lines of care is obvious in Iran. Pharmacists certified within this specialty are capable of optimizing and managing the medications for patients with complex needs and who are also ambulatory. This is a ground-breaking step for pharmacists in Iran, as it will bring together the two most important aspects of pharmacy practice, which are medicines knowledge and accessibility, and solidifies a patient-pharmacist relationship historically reserved for physicians. Such initiatives enable the efficient use of healthcare providers to their maximum capabilities, with pharmacists focussing on patient/medicines management, while physicians can concentrate on diagnosis and treatment. Collaborative practice arrangements enable even more synergy among healthcare professionals in the care of complex patients.

With the aims of advocating increasing roles for pharmacists in the management of complex patients, and providing an extensive platform for learning and growth to do just that globally, International Pharmaceutical Federation (FIP) has made “Complex Patients” a priority for 2013 and will examine the issue from all standpoints: biological (emphasising the current development of systems biology), medical (demographics, genetics, smoking, alcohol, diet and multiple diseases), socio-economic (availability of resources, and literacy) and cultural (beliefs, traditions, and religion). Pharmacists have the ability and opportunity to support patients in every aspect of complexity. 

Emphasis should be make on the fact that patients are likely to become increasingly complex as they grow older in the Iranian society, and develop multiple diseases requiring treatment with several medicines. In turn, this creates the need for integrated care across medical specialties and effective collaboration within a team of health professionals. The pharmacist is an important member of this team, with an important role to play in understanding and managing the complex patients, especially with respect to responsible medicines use.

It is imperative that complexity is also considered from the perspective of the patient, who may or may not consider themselves “complex”. For each person, their primary concern is how their illness, and secondary to that, their medicine are taking care. Dr Timothy Chen, associate professor at the University of Sydney specialising in mental health, says that it is known, for example, that pharmacists often feel more comfortable and confident contributing to the management of physical conditions, such as cardiovascular disorders, than mental disorders. He explains that the global disease burden arising from mental illness is immense. Although there are different modes of management for mental disorders (*eg. *psychotherapy), drug therapy is the major modality of treatment for many conditions, such as depression, bipolar disorder and schizophrenia. Therefore pharmacists, as experts in pharmacotherapy, should have a major role in the management of mental illness. 

Equally, there are many other factors that can make the management of patients with a mental illness more complex. Examples include the high rate of medication non-adherence (estimated to be approximately 50%) in patients prescribed antidepressant and other psychotropic medicines and the burden of managing significant adverse effects, such as diabetes, weight gain and dyslipidaemia. Although there are many challenges in the management of mental illness, pharmacists should and do have a major role to play in the delivery of effective health care. 

Pharmacological management in these circumstances is challenging, with a host of issues including the number of medications required, the risk of drug-drug interactions and the need to minimise drug toxicity. 

Nowhere do pharmacists interact with complex patients more than in the community. Pharmacists should be at the core of partnerships when it comes to managing diseases in the community such as GI disorders, high blood pressure, asthma, monitoring patients, performing triage, advising about treatment options and preventing additional complexities. 

Although a great deal of action has taken place with the incorporation of clinical pharmacy course in the general pharmacy education curriculum, but a serious demand is obvious to take action for in-practice pharmacies with the arrangement of continues education programs with headings such as; why patients are complex, needs of complex patients, how these needs are currently being met, and emerging and future strategies for treating the complex patients. By providing evidence-based scientific information and embracing collaborative practice, the pharmacist should have a critical role in dealing with complexity in patient care.

*Farshad Hosseini Shirazi is currently working Professor at the Pharmaceutical Sciences Research Center and Department of Toxicology and Pharmacology, Faculty of Pharmacy,Shahid Behesthi University of Medical Sciences, Tehran, Iran. He could be reached at the following e-mail address:* fshirazi@yahoo.com. 

